# The Role of Cardio-Renal Inflammation in Deciding the Fate of the Arteriovenous Fistula in Haemodialysis Therapy

**DOI:** 10.3390/cells13191637

**Published:** 2024-10-01

**Authors:** Jamie Kane, Alaura Lemieux, Gaurav Baranwal, Sanjay Misra

**Affiliations:** Vascular and Interventional Radiology Translational Laboratory, Department of Radiology, Mayo Clinic, 200 First Street SW, Rochester, MN 55905, USA; kane.jamie@mayo.edu (J.K.); lemieux.alaura@mayo.edu (A.L.); baranwal.gaurav@mayo.edu (G.B.)

**Keywords:** vascular smooth muscle cells, inflammation, arteriovenous fistulas

## Abstract

Vascular access is an indispensable component of haemodialysis therapy for end-stage kidney disease patients. The arteriovenous fistula (AVF) is most common, but importantly, two-year failure rates are greater than fifty percent. AVF failure can occur due to a lack of suitable vascular remodelling, and inappropriate inflammation preventing maturation, or alternatively neointimal hyperplasia and vascular stenosis preventing long-term use. A comprehensive mechanistic understanding of these processes is still lacking, but recent studies highlight an essential role for inflammation from uraemia and the AVF itself. Inflammation affects each cell in the cascade of AVF failure, the endothelium, the infiltrating immune cells, and the vascular smooth muscle cells. This review examines the role of inflammation in each cell step by step and the influence on AVF failure. Inflammation resulting in AVF failure occurs initially via changes in endothelial cell activation, permeability, and vasoprotective chemokine secretion. Resultingly, immune cells can extravasate into the subendothelial space to release inflammatory cytokines and cause other deleterious changes to the microenvironment. Finally, all these changes modify vascular smooth muscle cell function, resulting in excessive and unchecked hyperplasia and proliferation, eventually leading to stenosis and the failure of the AVF. Finally, the emerging therapeutic options based off these findings are discussed, including mesenchymal stem cells, small-molecule inhibitors, and far-infrared therapies. Recent years have clearly demonstrated a vital role for inflammation in deciding the fate of the AVF, and future works must be centred on this to develop therapies for a hitherto unacceptably underserved patient population.

## 1. Introduction

Chronic kidney disease (CKD) is a serious public health issue in the United States, causing progressive loss of kidney function [1]. CKD is defined in terms of the estimated glomerular filtration rate (eGFR) and/or albuminuria where eGFR has fallen below the threshold value of 60 mL/min/1.73 m^2^ and albumin levels are greater than 30 mg/g [2]. In 2023, 14% of US adults had a CKD diagnosis. Most people with CKD do not progress towards end-stage kidney disease (ESKD), where eGFR falls below 15 mL/min/1.73 m^2^, but some 2219 cases of ESKD per million Americans were reported in 2021 [1]. Kidney replacement therapies are considered for people with continuously progressive ESKD, and in the absence of readily available transplant options, the main modality chosen to extend their life is haemodialysis therapy (HD) [3]. This therapy involves the haemodialysis machine filtering the blood. The system has two circuits, which never directly meet, a blood circuit and a dialysate circuit. The connection between the two sides is via the dialyser, which comprises a dialysing membrane which filters out uraemic toxins. Most often, this technique is performed in-centre and requires 3–4 sessions per week [2,4].

Vascular access must be created to enable haemodialysis therapy, and this can be in the form of an arteriovenous fistula (AVF), arteriovenous graft (AVG), or central venous catheter (CVC) [5]. AVFs are the most common choice where 60% of prevalent American adults receiving HD do so via an AVF access [1]. Utilising an AVF is favourable because of longer-term patency and fewer vascular events like thrombosis, stenosis, and infections as compared to AVGs or CVCs [6]. Before the AVF can be used, it must be mature, meaning that it is easily cannulated while providing sufficient blood flow and having low flow resistance—establishing these characteristics is a complex and multi-faceted process. However, all three vascular access methods are highly prone to complications impinging on the efficacy of HD [7].

### AVF Complications and Failure Are Common in Haemodialysis

**Figure 1:**
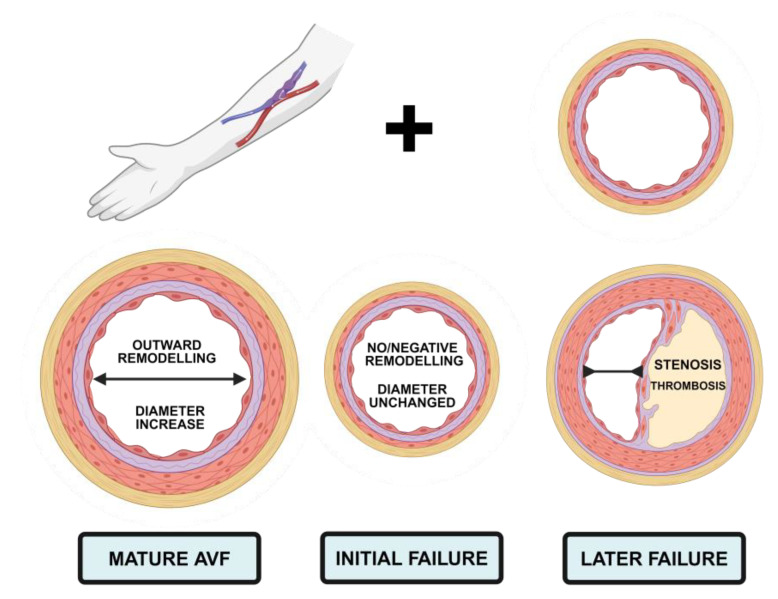
Suitable vascular remodelling determines initial AVF maturation and long-term patency. Following AVF creation, the vessel must thicken and remodel outwardly, increasing the internal diameter to allow for appropriate haemodialysis. Failure immediately following surgery involves no vascular remodelling or increases in diameter. Alternatively, failure over a longer term typically involves venous stenosis secondary to neointimal hyperplasia. Made with BioRender.

Despite AVF creation being the preferred choice in HD, AVF failure is common, and the cumulative two-year loss of primary unassisted patency is at 47.3% [1]. Non-maturation, thrombosis, stenosis, or eventual failure can occur in the absence of appropriate vascular remodelling [8]. Percutaneous transluminal angioplasty (PTA) is considered the gold standard for treating AVF stenoses and is indicated once the reduction in lumen size begins to affect dialysis efficacy [5]. However, even with these treatments, restenosis is common and the AVF often still fails [9,10]. Subsequently, further access points (possibly of other access types) must be created, or the person may be transferred to other dialysis modalities like peritoneal dialysis [11]. The maturation or the failure of an AVF can be thought of as a balancing act between desired vascular responses and several failure risk factors and can be delineated to two phases—deleterious vascular remodelling in the maturation phase and unwanted vascular responses to ongoing dialysis [12,13,14]. Following surgical creation, AVF maturation depends on an appropriate response to the intervention with a progressive increase in arterial and venous diameters and subsequent blood flow. These changes are, in successful AVFs, a suitable response following haemodynamic changes secondary to the anastomosis such as increased blood flow, blood pressure, and shear stress [15,16,17]. Suitable vascular responses to these injuries are the release of endothelium-derived vasodilators such as nitric oxide (NO) and prostacyclin. These molecules promote increases in luminal area, block thrombus formation, and promote vascular smooth muscle cell (VSMC) differentiation and transmigration. Subsequently, NO interacts with the matrix metalloproteinases 2 and 9 (MMP-2, MMP-9) and promotes further dilation [18]. Consequently, desirable outward structural remodelling occurs with elastin fibre degradation, VSMC hypertrophy, and realignment of endothelial cells (ECs) to the new flow direction [16,19,20]. These changes must take place without compromising the luminal area, as shown in Figure 1. In the context of non-maturing AVFs and later AVF failure, one or several of these processes fail, resulting in venous neointimal hyperplasia (VNH), unfavourable remodelling, and subsequent decreases in luminal area beyond a point of suitability for HD therapy [21]. Vascular remodelling is required for AVF maturation, but the processes carry with them risk for AVF failure as do subsequent inflammatory events induced by HD itself like repeated needling [22]. Inflammatory burden associates with AVF failure. The precise molecular mechanisms underpinning AVF failure are complex and poorly understood. A substantial part of AVF failure is the postulated association with the inflammatory state [23,24,25], which will form the focus of this review. In people undergoing AVF creation, interleukin-6 (IL-6) serum levels [26], C-reactive protein (CRP) levels [27], IL-1β, and C-X-C chemokine receptor type 4 [28] (CXCR-4) were significantly increased in those with dysfunctional versus functional fistulas. Additionally, increases in the neutrophil-to-lymphocyte and platelet-to-lymphocyte ratio are also strongly predictive of AVF maturation [29,30,31]. Relatedly, throughout several animal studies in our lab [23,24,32,33] and from other groups [34,35,36], the essential role of inflammation in controlling the development and maturation of the AVF has become clear, as has the pivotal role anti-inflammatory therapeutics may play. Therefore, this review will describe and discuss the impact of inflammation on each of the cell types involved in AVF failure, and then go on to discuss how therapeutic intervention in these therapies may be beneficial and what they can reveal about the mechanistic underpinnings.

## 2. The Role of Inflammation in Each Cell Type in the Cascade of AVF Failure

### 2.1. Endothelial Cells

The endothelial layer is incumbent in two important roles—managing the infiltration of immune cells into the vessel walls and offering VSMC-enhancing delivery of nitric oxide (NO). Firstly, the degree and scale of endothelial dysfunction in the early stages of AVF development predict future VNH and VS. Specifically to the juxta-anastomotic site, there is an early increase in the endothelial permeability and expression of vascular cell adhesion molecule-1 (VCAM-1) [37], which is then strongly correlated to the greatest degree of stenosis [37]. More broadly, impairment of endothelial function associates with less remodelling, a reduced venous diameter, and a reduced rate of successful AVF maturation [38].

This includes microvascular dysfunction in addition to the established macrovascular dysfunction of the AVF itself. Korsheed et al. already described some 10 years ago how the creation of an AVF associates with both local and remote changes in the microcirculation, as assessed by laser doppler and endothelial-dependent vasodilation assays [39]. The exact changes remain unclear; however, a recent study found no change in microvascular endothelial function from baseline up to 6 weeks after AVF creation, but a better baseline microvascular function did associate with a better probability of successful AVF maturation [40]. The major changes induced by the haemodynamics of the AVF, and the trauma involved in its creation, may therefore be inducing inflammation in distant microvasculature sites, leading to further potentiated inflammatory responses.

The other role for ECs is the delivery of NO. Mechanistically, endothelial injury, such as that induced by TNF-β secondary to repeated needling, results in mitochondrial reactive oxygen species (ROS) generation [41,42,43]. As a result, there is reduced endothelial NO production and less vasodilation. A recent study from the Lee group described the protective effects of eNOS overexpression on the natural development of the AVF. The overexpression of eNOS was able to modify the flow dynamics in the AVF, and the presence of more eNOS reduced wall shear stress and vorticity as compared to wild-type mice and eNOS knockout mice [44]. The modulation of EC function and NO delivery may therefore be an approach to improve response to physical stressors of haemodialysis and in the inflammatory cascade secondary to AVF creation and ongoing uraemia.

These effects may be modifiable directly via the modulation of the vitamin D receptor (VDR) [33,45]. VDR expression was strongly decreased in venous tissue samples of patients with AVF stenosis, whilst the expression of P66Shc, P-P66Shc, fibronectin, collagen-1, and 8-OHdG were increased significantly. These findings were mirrored with concomitant increases in mitochondrial ROS species, following the stimulation of HUVECs with TGF-β. Relatedly, in this in vitro model, the overexpression of VDR and the modulation of Pin1, a regulator of P66, rescued TGF-β-induced injury. Therefore, the modulation of VDR signalling may be a new therapeutic avenue. Improvements to endothelial function may be directly achievable by the administration of vitamin D. Specifically to CKD, human endothelial cells cultured in a CKD-like environment showed reduced inflammatory cytokine levels and increased eNOS expression following vitamin D3 administration [46]. In vivo, uraemic rat paricalcitol administration was able to attenuate endothelial dysfunction by restoring cell–cell contacts and reducing permeability, a finding also mirrored in human endothelial cell lines [47].

Similarly, in a recent study from our own group specifically on the AVF context [33], we administered 1α, 25 (OH)_2_D_3_ encapsulated in nanoparticles to the adventitia of a surgically created AVF in uraemic mice and pigs [33,48] and then after a PTA was also performed on the porcine AVF [49]. This treatment reduced the presence of the key immune markers MCP-1 and CD68 in the outflow vein. Blood flow and lumen area were significantly increased whilst maximal velocity, VNH, and shear stress were reduced. In line with the previously discussed studies, RNA sequencing revealed an upregulation for TGFβ1 pathways. Taken together, these data support an essential role for the vascular endothelium, possibly modulated by VDR expression.

Patients receiving haemodialysis very often have several comorbidities, which may predispose them to endothelial injury and a predication for therefore problematic vascular rebuilding following AVF creation. A major comorbidity is atherosclerosis [50], which is triggered in its initial stages by endothelial dysfunction [51,52] and accelerated in dialysis [50,53]. The causes and pathobiology are beyond the scope of this review, but atherosclerotic endothelia are activated and express high levels of adhesion molecules to facilitate an easy transmigration of immune cells [54]. These processes have clear parallels to the outlined process of AVF failure and may be interrelated, particularly given how inflammation in chronic disease may be one and the same [55]. Vascular calcification is the other major comorbidity in people receiving haemodialysis therapy [56]. During pre-operative screening, the surgeon aims to select against calcified arteries, but given the close-to-universal prevalence of some cardiovascular calcification in HD patients [57], there are still up to 23% of AVFs that have calcifications [58]. A preliminary event in calcification forming is the over-formation of calciprotein particles (CPPs), which have recently been shown to impair endothelial function. CPPs reduce NO bioavailability via reducing the expression of eNOS, leading to increased exposure to reactive oxygen species [59,60]. Taken together, the baseline state of the vessels of a patient in which an AVF is created may itself influence resulting AVF failure due to endothelial dysfunction and inflammation.

Recently, there was an association described between the atherosclerotic risk score and AVF failure, identifying this as a potential new risk score, but also supporting the role for a tendency toward endothelial dysfunction in driving unwanted vascular outcomes. Despite the best intentions of the surgeon, pre-existing atherosclerotic changes in the target vessel, underpinned by endothelial dysfunction, may influence AVF failure [61]. Separately to the main AVF itself, the microvasculature may play a role; a recent study found microvascular endothelial function to be predictive of AVF success and subsequent function, possibly a supporting determinant of the ability to dilate in response to new shear stress following surgery [62].

Endothelial damage may also be due to the process of performing dialysis itself. In addition to the turbulent stress the endothelium experiences due to surgery, repeated needling is associated with endothelial damage. This needling can activate the endothelium, potentiate dysfunction, and lead to downstream failure and damage to the AVF from such direct physical hits [63]. Lastly, the uraemic milieu experienced by those receiving haemodialysis can be responsible for further endothelial dysfunction. Cell viability, proliferation, migration, and healing capacity are negatively impacted by uraemic serum [64,65,66,67], which in turn impinge upon suitable endothelial function. A downstream result may be a disposition to allow immune cell infiltration from the circulation to the subendothelial space. Infiltrating immune cells and their behaviour are the second lynchpin in deciding the fate of an AVF.

### 2.2. Immune Cell Infiltration

The exaggerated process of neointimal hyperplasia inside a vessel is a complex process initiated by endothelial damage, exposing the vascular smooth muscle cells to the blood components. This complex process is further affected by the proliferative and inflammatory responses [68]. In this section, we will elaborate upon different immune cells and how their infiltration affects the progression of the VNH. Immune cell infiltration in AVF progression can be divided into three distinct categories: firstly, the initial inflammation necessary for AVF maturation; secondly, the unchecked runaway inflammation causing the failure of AVF; and finally, the therapeutic strategy targeting the inflammation, which results in VHS/VS.

### 2.3. AVF Maturation

The histological findings from the initial creation of AVF are clearly indicative of inflammatory cell infiltration as early as 4 h, which attenuates vein graft endothelial cells and elevates subendothelial oedema [69,70]. Kuwahara et al. reported that M2 macrophage infiltration predominantly supports the extracellular matrix (ECM) deposition (hyaluronic acid) required for AVF maturation mainly via interaction with a widely expressed cellular adhesion molecule, CD44. This ECM:CD44 interaction supports the adhesion of leukocytes to endothelial cells, inducing the macrophage secretion and regulating VSMC proliferation and migration [71]. Other studies have also suggested that the role of M2 macrophages is important in vascular wall thickening and supporting the AVF maturation. The important immune cells that help in facilitating an adequate blood flow and guiding the outward remodelling are the CD4+ T cells [72,73,74]. Despite the fact that immune infiltration helps in vascular modelling, there needs to be a balance between pro-inflammatory and anti-inflammatory cells/response as both excessive and insufficient responses lead towards AVF failure [25].

### 2.4. AVF Failure

AVF failure is attributed to one of two stages, either early-stage failure linked with immaturity of AVF or later-stage/chronic failure mostly due to uncontrolled immune responses, leading to negative remodelling and reduced patency of AVF [13]. The pathological steps around adventitial remodelling have been reported to start with the infiltration of myofibroblasts into the adventitia [75,76], while further reports suggest endothelial-to-mesenchymal transitions (EndoMETs) as the main phenomenon involved, leading towards the fibrotic vessel. These mechanisms mainly are driven by the activations of signalling cascades associated with PI3K/Akt/mTOR pathways. These signalling pathways are activated by several cytokine/growth factors such as TGFβ, which is secreted by T cells and macrophages. The role of macrophages and T cells is not yet defined clearly as they play a role in both maturation (positive remodelling) and failure (negative remodelling) [30,72,74,77]. Samra et al. investigated the major immune cells by IHC and qRT-PCR, in the miniswine model, to further add to the strong association of immune response in AVF creation and maturation [78]. Our group also composed a review earlier about various vascular molecular messengers associated with haemodialysis vascular access malfunctions at early local inflammation and later a systemic rise in inflammation, which helps us to lead and design different therapeutic approaches to increase the patency of AVF by targeting these inflammatory cells/cytokines [79,80,81,82].

### 2.5. Therapeutics

The research work from our lab and other groups has established the strong role of macrophages in the progression of the VNH/VS. Blocking CX3CR1, an active player involved with macrophage survival and migration, significantly reduced neointima with increased lumen vessel area and patency in a mice model of AVF [23]. In another study, genetic deficiency of MCP-1 enhanced fistula patency by 6 weeks after its formation with reduced venous cell wall thickness and increased luminal area in MCP1−/− compared with MCP-1 +/+ mice [83]. A transcriptomic study identified a role for differentially expressed genes like tripartite motif-containing protein 55 (TRIM55), myoblast determination protein 1 (MYOD1), and several other genes. These play a crucial role in inflammation and the regulation of inflammatory pathways, and immune regulation has strong association with vascular cuffing and chronic inflammation-mediated early thrombosis in AVF [84]. A bioinformatic study identified significant elevation in PTGS2 in AVF failure. In a murine AVF model, a selective inhibitor of PTGS2 significantly altered different immune cells and the most significant ones had a positive correlation coefficient with mast cell activation, and the highest negative correlation coefficient was observed for resting mast cells [85]. The role of matrix metalloproteinase has been well established in the maturation and failure of AVFs. In an MMP-9 knockout mouse model, AVF fistula stenosis was attenuated mainly by reducing perioperative vascular inflammation [86]. TNFα is another key regulator of inflammation, and drugs targeting TNFα have been well studied in several diseases; however, they have not yet been approved to treat vascular stenosis [87]. Future studies are needed in appropriate study models to define the definitive subtype of macrophage (M1/M2) and T cell (Tregs/Th1/Th2/TH17) roles in both AVF maturation and AVF failure to effectively target the specific immune regulators and thus enhance the patency of the AVF.

## 3. VSMCs

Vascular smooth muscle cells (VSMCs) are an essential component of the vascular system. VSMCs are located intervascularly in the medial layer and are essential for contraction, vessel stability, and consistent blood pressure to allow transport of blood and other cell types. Mature VSMCs can dedifferentiate unlike most cells in a vascular setting [88,89,90,91,92,93,94]. This dedifferentiation is identifiable by protein markers of α-smooth muscle actin and calponin, which are needed to continue a contractile state [95]. The dedifferentiation will not occur unless triggered by injury or illness [93,95,96,97].

In the absence of injury, the cells stay in a contractile state [95]. This contractile state is where proliferation is lowest, and the cells continue to express the protein markers α-smooth muscle actin and calponin [95]. Whilst these proteins continue to be expressed, low proliferation will continue until dedifferentiation begins.

The protein levels of SM22 and alpha smooth muscle actin (αSMA) remain low as dedifferentiation has not occurred. Once dedifferentiation occurs, these proteins are secreted [95,98]. Endothelial cells (ECs) and VSMCs are mutually supportive and assist in the continual support throughout the vascular system, offering enhancing functions for the other. ECs create a contractile state for the VSMCs and secrete glycosaminoglycans; this includes heparin and other heparin-like substances [95,98]. The ECs will continue to excrete the substance until an injury occurs. When an injury occurs, the ECs stop secreting the substance and allow the VSMCs to inundate the wound site. The state of the VSMCs evolves when glycosaminoglycan ceases; this new state is called a synthetic one [89,91,92,93,95,98]. The synthetic state is where the most proliferation and cell differentiation take place for VSMCs that have dedifferentiated from the contractile state [89,91,92,93,95,98]. After the VSMCs have started to dedifferentiate into a synthetic state, an opening has occurred that allows macrophages to infiltrate into the cell wall and affect how the smooth muscle cells interact with each other [89,91,92,93,95,98].

### 3.1. VSMC Dysfunction in AVF Inflammation and Failure

VSMCs are known to have crosstalk between ECs, immune cells, and macrophages, which allows for research to be performed on what cell or cytokine causes specific changes in phenotypes of the smooth muscle cells [89,94,95]. Rai et al. studied the ability of VSMCs to crosstalk and how that could affect atherosclerosis maturation and plaque growth. They also investigated what cells are involved before and after VSMC phenotype switching. Rai et al. found that changing the cytokines downstream of where phenotypic switching occurs can change how the VSMCs are modulated. The authors state that future studies on immune cell infiltration will show a decrease in inflammation and mitigate the damage being done to AVFs as they mature.

In one study, Bezhaeva et al. looked specifically at TLR4 in response to inflammation in a murine AVF model [99]. As TLR4 is a common precipitator of inflammation in microbial infection, atherosclerosis, and vascular remodelling, they targeted a known regulator on inflammatory immune cells and VSMCs called RP105 (also known as CD180). The study allowed them to see how RP105 knockdown affected the αSMA levels immunobiologically in an AVF. They saw a marked decrease in αSMA between a wild-type mouse and an RP105−/− mouse [99]. They also saw a marked change in the lumen area and number of VSMCs located in the intima. As the VSMCs have a similar regulatory factor, TLR4, as an immune cell, removing RP105 affected them both in similar ways [99].

After AVF placement, studies have shown that VSMCs polarise to a synthetic phenotype, and extensive smooth muscle cell proliferation can occur [89,91,92,93,95,98]. The VSMCs can migrate to the location of injury and create a pro-inflammatory setting. Such a setting can cause a number of complications to an AVF’s long-term usage, including stenosis, oedema, thrombosis, and eventual AVF failure [94]. This migration is activated when the TGF-β pathway changes due to stress and the overall injury [92]. The changing in the VSMCs after their movement from the medial layer into all layers of a cell causes an oscillating effect. This oscillating effect has the VSMCs reacting to the changes in metabolism and inflammation, and the proliferation of more synthetic cells occurs due to the continual change in TGF-β [92].

This change creates a pro-inflammatory type of synthetic VSMC that interacts with ECs and allows for negative effects on the AVF site [92,94,98,100]. This synthetic state straddles a thin line between helpful and detrimental to any injury to the vascular system. More research into the versatile status of VSMCs is needed for long-term success of many types of vascular intervention. Research is currently being performed on metabolism [92,95], hormones [95,100], uraemia levels [96], and suppression [88,91,94]. Finally, there may be a crucial sex-based difference determining the fate of the AVF. Female AVFs have a decreased expression of BMP7 and IL17R and increased TGFβ-1 and its receptor [101]. Similarly, in the uraemic mouse with an AVF model, female mice had more negative vascular remodelling, reduced peak blood flow velocity, loss of proliferation, and increased apoptosis [101]. These sex differences should be investigated further to expand on their clinical impact and relevance.

### 3.2. Non-Inflammation-Based Factors Controlling AVF Failure

Whilst the focus of this review has been on cellular inflammation, there are other factors controlling the success of the AVF, which will be briefly discussed. The age of the patient is an important consideration, as typically older people have even more comorbidities than the general ESKD population [1], and represent the fastest growing haemodialysis group. Broadly, however, increasing age in and of itself does not lend itself to worsened AVF outcomes; rather, it is the relationship between age and other relevant comorbidities like the aforementioned atherosclerosis and vascular calcification that may drive increased failure rates [102,103,104]. The US Renal Database shows that once these hurdles are managed, the actual AVF failure rate is comparable between age groups [1]; what remains clear, however, is that older patients must have a unique specific treatment plan and approach to optimise their outcomes. Sex of the patient may play a role, as females are at a greater (2.42x) risk of AVF failure compared to males [105]. US patient statistics show a somewhat mixed state, as females have higher overall rates of fistula maturation and yet the duration of patency is shorter as compared to males [1]. The precise etiological reasons for this discrepancy are beyond the scope of this review but must be considered.

The Dialysis Outcomes and Practice Patterns Study reported data from 12 countries and determined that surgical prowess and experience was a major factor determining eventual AVF success. The more AVFs a surgeon had already placed was directly correlated to the maturation rate of the placed AVF. In nations or regions where the training for doctors focused more on vascular access, the eventual outcomes for AVFs were strongly improved [106]. Other socioeconomic factors may have a mixed effect on the outcomes of AVFs, and patients from a lower socioeconomic background may have restricted access to healthcare and are less likely to receive an AVF and when they do, the outcome is worse [107], but these findings are not consistent across nations; the National Health Service in the UK does not report these changes [108], and similar findings in the US vary between some effect and no effect of the socioeconomic group [109,110]. Taken together, there is a wide complicated collection of factors controlling and modulating AVF success or failure, which vary nation to nation and centre to centre. Therefore, in seeking to ameliorate the burden placed by AVF failure, the focus must be placed onto universally treatable factors, which ideally involve therapies aimed at easing inflammatory burden locally in the AVF and systemically.

### 3.3. Therapeutic Insights and Progress

The advancements in knowledge and insight we have so far outlined have generated several new lines of inquiry in developing effective methods to treat and prevent stenosis and ease the burden on people with ESKD. As recently as 2006, the vascular access guidelines from the national kidney guidelines did not address pharmaceutical intervention to resolve access failure, instead relying on PTA or surgical relocation [111]; beneficially, there are now some emerging therapies of choice [5]. Far-infrared therapy (FIR) has in recent years found favour in abrogating inflammatory responses in the AVF. The mechanism remains unclear, but the non-thermal effects are likely via modifying the oxidative state and subsequent inflammatory milieu. FIR increases the expression of hemoxygenase-1, resultingly increasing antioxidant levels, reducing the TNF-α-induced expression of endothelial adhesion molecules, and restoring endothelial nitric oxide synthase and subsequent nitric oxide levels and endothelial function. FIR is seemingly efficacious in patient groups at each stage of the HD process, in those patients with a high HD vintage and several interventions on pre-existing AVFs [112], in patients with a high HD vintage but no interventions [113,114], and in those new to HD with a new AVF [115].

**Figure 2:**
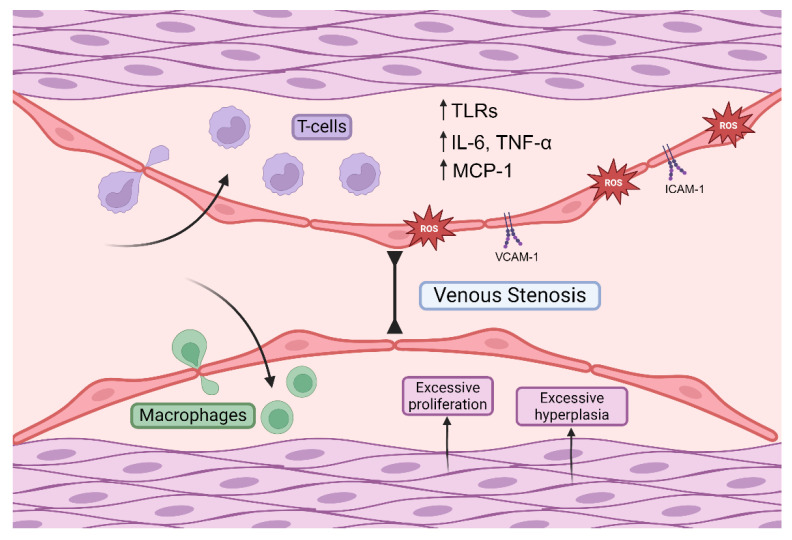
Uncontrolled runaway inflammation underpins and drives the failure of the arteriovenous fistula. Inflammation in the AVF involving the endothelial layer, subsequent immune cell infiltration, and cytokine changes therein, and smooth muscle changes are shown. These are essential processes in determining the fate of the arteriovenous fistula. Reactive oxygen species (ROS). Toll-like receptors (TLRs). Tumour necrosis factor-alpha (TNF-α). Monocyte chemoattract protein-1 (MCP-1). Interleukin-6 (IL-6). Vascular cell adhesion molecule-1 (VCAM-1). Intercellular adhesion molecule-1 (ICAM-1). Made with BioRender.

A more recent study by Lindhard [116] and colleagues draws this into question at least with a single treatment course where the vasoprotective effects could not be seen, but changes in soluble VCAM and ICAM were present. Whilst an emerging and promising choice [117], FIR is clearly in need of much more work before it can be developed into a potent clinical tool.

Small-molecule inhibitors have found favour—in a study from our own group, we demonstrated that a nanobody blockade of CX3C motif chemokine receptor 1 (CX3CR1) in a murine AVF model reduced neointima formation and increased lumen vessel area in the outflow vein. These changes were associated with reductions in CD68 and CX3CR1 expression in both mice and human samples [23]. In experimental murine AVF, a week of 1.14 mg/kg/day of atorvastatin potently decreased venous outflow inflammation [82], likely by reduced macrophage infiltration and fibrin deposition [118], and statin use in humans is associated with an 18% reduction in the risk for AVF failure [119]. Lastly, a retrospective cohort study predicted utility for statins in AVF treatment following creation [120]. The reliability in these findings may be doubtful; however, a more recent randomised clinical trial found no efficacy in maintaining AVFs following treatment with rosuvastatin [121], but importantly the trial was underpowered, finished prematurely, and only utilised a low dose—so a follow-up study is essential to confirm these findings.

Lastly, mesenchymal stem cells have emerged as promising candidates for modulating inflammatory responses in the AVF. Firstly, we treated mouse AVFs at the time of surgical creation with human adipose tissue-derived mesenchymal stem cells (MSCs) and saw reduced intimal hyperplasia and MCP-1 expression [122]. These findings were similar when MSCs were administered at the time of PTA instead; there was less IL-1β and TNF-α, fibrosis, proliferation, and smooth muscle actin deposition [123]. These changes were further associated with improved haemodynamics, with increased blood flow and lumen vessel area and reduced neointima [123]. Currently, the MEST AVF trial is ongoing to determine if this approach can extend into patient groups [124].

## 4. Conclusions

There is now a large pool of experimental evidence supporting a key and indispensable role for inflammation in deciding the fate of an AVF. These changes are a complex interplay between the local environment, individual cell types, the interactions between them, the patient, and the wider clinical setting. Despite a mounting collection of evidence describing AVF failure secondary to inflammation which are summarised in Figure 2, there is still an unacceptably sparse number of therapeutic options available. Investigating and developing the more promising solutions must become a key research focus in the coming years to ease the burden of AVF failure in haemodialysis therapy.

## Data Availability

No new data were created or analysed in this study. Data sharing is not applicable to this article.
